# Profiling of Hepatocellular Carcinoma Cell Cycle Regulating Genes Targeted by Calycosin

**DOI:** 10.1155/2013/317926

**Published:** 2013-12-23

**Authors:** Dongqing Zhang, Shufang Wang, Liguo Zhu, Yaping Tian, Haibao Wang, Yuan Zhuang, Yu Li, Deqing Wang

**Affiliations:** ^1^Department of Blood Transfusion, General Hospital of PLA, Beijing 100853, China; ^2^Department of Toxicology, Beijing Center for Diseases Control and Prevention, Beijing 100013, China

## Abstract

We cocultured calycosin with human hepatocellular carcinoma cell line (BEL-7402) to investigate the effect on cell proliferation. Calycosin can markedly block the cell growth in G_1_ phase (*P* < 0.01) on the IC_50_ concentration. There were seventeen genes involved in cell-cycle regulation showing differentially expressed in treated cells detected by gene chip. Eight genes were upregulated and nine genes were downregulated. Downregulated TFDP-1, CDKN2D, and SPK2 and upregulated CDC2 and CCNB1 might affect cell cycle of tumor cells. Furthermore, we checked the transcription pattern using 2D gel method to find different expression of proteins in human hepatocellular carcinoma cells after exposure to calycosin. Fourteen proteins were identified by matrix-assisted laser desorption/ionization-time of flight-mass spectrometry (MALDI-TOF-MS). Twelve proteins expression were increased such as transgelin 2, pyridoxine 5′-phosphate, stress-induced-phosphoprotein 1, peroxiredoxin 1, endoplasmic reticulum protein 29, and phosphoglycerate mutase 1. Only thioredoxin peroxidase and high-mobility group box1 proteins' expression decreased. Both genes and proteins changes might be relate to the mechanism of antitumor effect under treatment of calycosin. In conclusion, calycosin has a potential effect to inhibit the BEL-7402 cell growth by inhibiting some oncogene expression and increasing anticancer genes expression, what is more, by blocking cell cycle.

## 1. Introduction

*Astragalus mongholicus* can improve body's specific and nonspecific immune functions, promote immunocytokine generating, and inhibit the growth of tumor cells. *Astragalus mongholicus* enhances immune function by increasing the activity of certain white blood cells which increases the production of antibodies (IgA and IgG), increases the production of interferon, and stimulates natural killer cells.

In addition to boosting immunity, *Astragalus* has antibacterial, anti-inflammatory, and antiviral effects. It contains numerous components, including polysaccharides, flavonoids, triterpene glycosides, amino acids, and trace minerals. Besides the main function of enhancing the NK cells activity, a series of studies also indicated the remarkable inhibition of flavonoids of *Astragalus* (TFA) on human hepatoma cell line BEL-7402 and a variety of leukemia cells proliferation in vitro [1, 2], and Xu Du-juan found that *Astragalus* significantly inhibited the growth of transplanted hepatoma (HepA) in mice and sarcoma (S180) and the growth of HeLa cells in vitro [3]. These studies denoted that the ingredients in *Astragalus* not only had the antitumor effect on the overall level but also had direct inhibition on tumor cell in vitro. Reported TFA also blocked the cell-cycle in G_0_/G_1_ phase, after incubating HeLa cells together for 5 days [3].

However, there were no reports about the inhibitory action of calycosin, one of the capital chemical compositions of TFA on tumor cells and the mechanism of anti-tumor. The calycosin was a main active ingredient of the *Astragalus* and liquorice [1]. In this study, neutral red (NR) assay, flow cytometry, gene chip detection, and two-dimensional gel electrophoresis were performed to investigate the effect of calycosin on human hepatocellular carcinoma cell line (BEL-7402), in an attempt discover some detailed mechanism. We found that calycosin can hinder BEL-7402 cells into S phase and inhibit tumor cell proliferating rapidly by blockage in G_1_ phase.

## 2. Materials and Methods 

### 2.1. Materials

Calycosin was isolated and identified by the Department of Biochemistry of Chinese People's Liberation Army (PLA) General Hospital. The kits of cell-cycle were offered by the company of BD. The gene chips of human cell-cycle were prepared and analyzed by the CapitalBio Corporation.

### 2.2. Culture of BEL-7402 Cells

Human hepatocellular carcinoma cell line (BEL-7402) was obtained from the Chinese Academy of Science Shanghai Institute of Cell Biology. BEL-7402 cells were cultured in RPMI1640 medium (GIBCO) containing penicillin and streptomycin and 10% inactivated fetal bovine serum under the conditions of 37°C, 5% CO_2_. When the cells were at the stage of logarithm growth, all the experiments were carried out.

### 2.3. Determination of the 50% Inhibitory Concentration (IC_50_) of Calycosin

The IC_50_ was measured by neutral red (NR) assay. The BEL-7402 cells in the stage of logarithm growth were added into 96-culture plate at the density of 1 × 10^4^ per well in 0.1 mL volume. Different concentrations of drugs were added (final concentrations were 0.004, 0.011, 0.035, 0.111, 0.352, and 1.113 mmol/L) after 24 hours of incubation, four duplicates in each group. After 24 hours of incubation, neutral red was added to a final concentration 50 mg/L. Staining solution was discarded after of being incubated for 2 hours under the condition of 37°C. Then to each well was added 100 *μ*L extract solution (volume fraction of 1% the acetic acid and 50% ethanol), settled at room temperature for 20 minutes. OD was determined in the wavelength of 570 nm by the ELISA instrument and the growth inhibited rate of cancer cells was calculated.

### 2.4. Determination of Cell-Cycle by Flow Cytometry

Each 25 cm^2^ culture flask was plated with cells at the density of 7.5 × 10^5^. Drugs were added at the concentration of the IC_50_. After 24 hours the cells were digested by trypsin and washed twice by PBS. Precooled 70% ethanol was then added to start fixation for at least 12 hours at 4°C. Fixed cells were washed twice by PBS. RNaseA was then added to the final concentration of 50 *μ*g/mL and incubated at 37°C for 30 minutes. Propidium iodide (PI) was added and the labeled cells were loaded to Model ABC flow cytometry analyzer for analysis.

### 2.5. Preparation of RNA

Total RNA was prepared from control cells and treated BEL-7402 cells using Trizol reagent. Cells were washed twice by PBS; then Trizol reagent was added (1ml Trizol for 5–10 × 10^6^ cell) to extract the RNA according to the standard operation. RNA was concentrated by the precipitation method of isopropyl alcohol, purified by the NucleoSpin RNA clean-up kit. The OD of the RNA was measured by the spectrophotometer and the quality of the RNA was checked by the denaturing formaldehyde gel electrophoresis.

### 2.6. Determination of Gene Expression of Cell-Cycle

The samples were labeled with fluorescence by the method of RNA labeling and then were air-dried after being purified. The labeled samples were dissolved in hybridization solution and hybridized at 42°C overnight. The samples were washed in the washing solutions 1, followed by washing solution 2. Hybridized gene chips were scanned after being dried using dual-channel laser scanner of LuxScan 10 K/A (CapitalBio Corporation). The images of gene chip were analyzed by image analysis software of CapitalBio (CapitalBio Corporation). Then the signal of pictures was changed into digital signal. The data in the gene chip were normalized by the Lowess method. At last, the differentially expressed genes were determined by the one-class method of SAM. The test was repeated twice.

### 2.7. Two-Dimensional Gel Electrophoresis and Collection and Analysis of Gel Images

The BEL-4702 cells were divided into two groups, one group which was treated with calycosin at the concentration of IC_50_ and the blank. Cell proteins were separated by 2DE method and then silver-stained.

Two-dimensional electrophoresis gel after being stained was scanned using gel-image scanner (Amersham Pharmacia Biotech Company) and digital image document was analyzed by software (Image Master 2D Elite 5.0) for obtaining the relative molecular mass Mr, pI, isoelectric point pI, and relative amount of proteins.

### 2.8. Mass Spectrum Identification of Differentially Expressed Protein Points

Protein dose for two-dimensional gel electrophoresis was added to 1 mg and then stained. Decoloration, trypsin enzymolysis in gel, and extraction of peptide were proceeded on after some differential protein points were excised; last peptide mass fingerprint spectrum (PMF) was analyzed using MALDI-TOF-MS. Evaluation of results reliability depended on the matching rate of peptides and sequence coverage rate of matched peptides on corresponding proteins.

### 2.9. Statistical Method

SPSS15.0 software was used for analysis IC_50_ was calculated by pro method (Probit). The statistical discrepancy between cell-cycles was examined by *t*-test. Cluster 3.0 software was used for gene expression, the method of hierarchical and average linkage for cluster analysis. Differential expression genes were analyzed for statistical significance on MAS (molecule annotation system).

## 3. Results

### 3.1. Examination IC_50_ of Calycosin on BEL-7402 Cell

**Figure 1:**
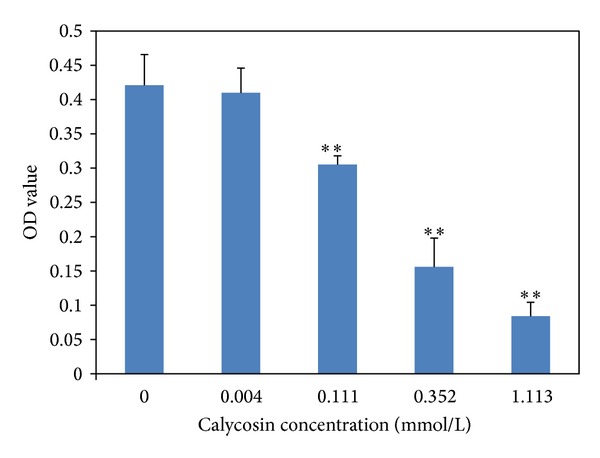
The effect of calycosin on proliferation of BEL-7402 cells.

The BEL-7402 cells were treated with calycosin for 24 hours in different dosages of calycosin as shown in Table 1. The inhibition of calycosin on hepatocellular carcinoma cell at concentration of between 0.035 and 1.113 mmol/L was shown as the significant dose-effect relationship. After the effect of calycosin on BEL-7402 for 24 hours, IC_50_ was 0.246 mmol/L by Probit analysis and the 95% confidence interval was 0.189–0.336 mmol/L (Figure 1).

Different concentrations of calycosin were added (final concentrations were 0.004, 0.011, 0.035, 0.111, 0.352, and 1.113 mmol/L), four duplicates every group, including blank and solvent control (0.5% DMSO). It shows that calycosin has a significant inhibitory effect on human hepatocellular carcinoma BEL-7402 cell in vitro. **means that *P* < 0.01 compared with control.

### 3.2. Calycosin Reduces the Proliferation of BEL-7402 Cells

**Figure 2:**
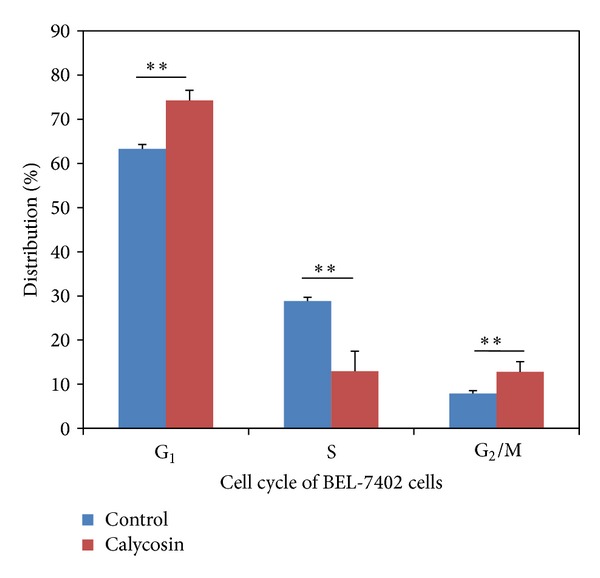
Effect of calycosin on BEL-7402 cell-cycle; **means that *P* < 0.01 compared with control.

Calycosin was added at the concentration of the IC_50_. After 24 hours the cells were digested by trypsin and washed twice by PBS. The labeled cells were loaded to Model ABC flow cytometry analyzer for analysis The calycosin could block the cell growth cycle in G_1_ phase significantly comparing with control group (Figure 2) causing remarkable decrease of S phase (Table 2).

### 3.3. The Effect of Calycosin on Gene Expression of Cell-Cycle and Check Points

**Figure 3:**
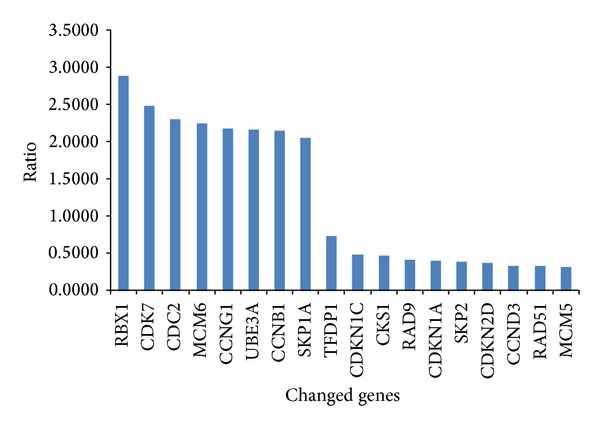
Effect of calycosin on cell-cycle genes expression of BEL-7402 cell.

The A260/A280 value of total RNA extracted from samples was between 1.60 and 1.75. In 10% agarose gel electrophoresis, the bands of 18S rRNA and 28S rRNA were clear and the band of 28S was the double of bands of 18S. The total RNA extracted could be used for chip hybridization. The probe marked with Cy3 (control) and Cy5 (calycosin) were hybridized with the 100 genes which were related to cell-cycle. 18 strips of differentially expressed genes were obtained (Figure 3); the ratio below 1 was regarded as downregulated gene and above 2 as upregulated gene. The upregulated and downregulated ratios of calycosin-treated/control were 3.602 ± 1.652 and 0.315 ± 0.128, respectively.

The data in the gene chip was normalized by the Lowess method. The differentially expressed genes were determined by the one-class method of SAM. The test repeated twice. 18 strips of differentially expressed genes was related to cell-cycle were obtained, 8 strips of expressed genes were upregulated, and 10 genes were downregulated.

### 3.4. Mass Spectrum Identification of Differentially Expressed Proteins

**Figure 4:**
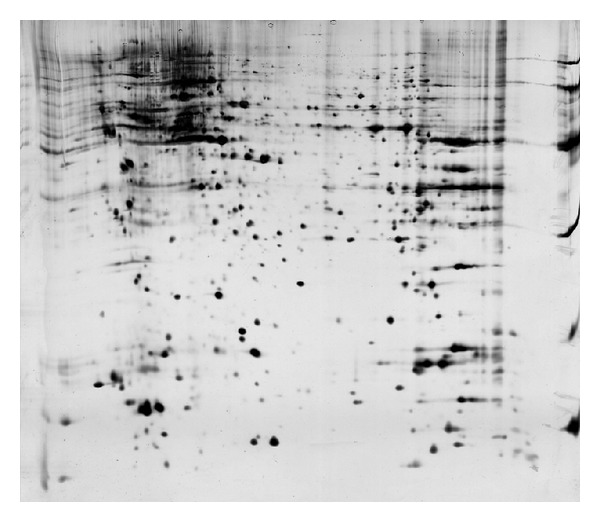
Differentially expressed proteins of human hepatoma carcinoma cells after the treatment of calycosin on 2DE gels.

One piece of 2DE gel could be divided into approximate 1200 protein points after being analyzed by Image Master 2D Elite 5.0 software. Repeated three times each group, the matching rate of same sample was more than 95 percent. The differentially expressed points marked on 2DE spectrum of a graph stained with coomassie brilliant blue (Figure 4), these on the drawing of partial enlargement between calycosin treated and control. 13 proteins for upregulated expression, 2 proteins for downregulated expression, and 3 proteins disappearance in calycosin-treated group were shown by 2-DE gels. The Mr and pI of differentially expressed protein points were shown (Table 3).

Two-dimensional electrophoresis gel after stained was scanned using gel-image scanner and digital image document was analyzed by software for obtaining the differently expressed proteins.

14 species of proteins were identified by mass spectrum (Table 4). Sites of some proteins did not match theoretical Mr and pI because of the posttranslational modification such as glycosylation and phosphorylation. 14 species of proteins including thioredoxin Peroxidase B, keratin 1, stress-induced-phosphoprotein 1 (Hsp70/Hsp90-organizing protein) isoform 1, abhydrolase domain containing 14B, pyridoxine 5′-phosphate oxidase, LIM and SH3 protein 1, peroxiredoxin 1, neuropolypeptide h3, transgelin 2, high-mobility group box 1, nonmetastatic cells 1, protein (NM23A) expressed in isoform, endoplasmic reticulum protein 29 isoform 1 precursor, phosphoglycerate mutase 1 (brain), and biliverdin reductase B were identified by mass spectrometric detection.

## 4. Discussion 

In our research, calycosin, one of major monocompounds extracted from TFA, was firstly demonstrated on the inhibition on growth of BEL-7402 cells in vitro. The proliferative ratio of tumor cells mainly depended on the length of G_0_/G_1_ phase and the ability of cell division [4]. We discovered that calycosin could mainly block growth of BEL-7402 cells in G_1_ phase cells in S phase causing to diminish markedly, after BEL-7402 cells being incubated with calycosin for 24 hours.

Flavonoids from plants can inhibit growth of tumor cell by regulating check points of cell-cycle [5–7]. Some studies had manifested that BEL-7402 cells had upregulated TFDP and E2F expression [8]. TFDP1 is a heterodimerization partner for members of the E2F family of transcription factors. E2F1/TFDP1 forms a complex involved in cell-cycle progression by the regulation of expression of cell-cycle promoters (cyclin A, cyclin E, and CDK2). TFDP-1 is one of the driver genes, which leads to higher tumor aggressiveness through deregulation of cell-cycle. Our results demonstrated that calycosin could downregulate the expression of TFDP1. In addition, abundant studies showed the high expression of SKP2 was one characteristic of human tumor growth [9–11], and SKP2 was not only used as an independent prognosis of cancer patients, but also as a new target of antitumor drugs and gene therapy [9]. In our results, calycosin affected two check points gene transcription; for example TFDP-1 and SKP2 were markedly downregulated and CDC2 and CCNB1 were upregulated expression and downregulated expression of CDKN2D which may block the G_1_/S and G_2_/M transition in cancer cells.

Also we tried to investigate the mechanism of calycosin in human hepatocellular carcinoma cell by screening different proteins expression in treatment groups. We identified 12 proteins expression increasing and 2 proteins expression decreasing. The upregulated expression of transgelin 2, pyridoxine 5′-phosphate oxidase, stress-induced phosphoprotein 1 (StIP1), peroxiredoxin 1, and biliverdin reductase B suggested that they played a key role in mechanism of calycosin antitumor function. Transgelin 2 protein expression is upregulated by calycosin. Previous studies show that transgelin 2 expression is decreased in some cancers. Transgelin 2 was also proved to have relationship with hepatitis B and liver carcinoma. The oncogene Ras inhibits the expression of transgelin 2 genes in thoracic wall and large bowel neoplasm and some other cancers [12–16]. The inhibition effect on tumor cell proliferation of calycosin might be caused by upregulated transgelin 2 protein expression. Calycosin can increase the PNPO protein expression. A series of researches indicated that PNPO could regulate cell proliferation and apoptosis [17], but lack in liver or neurogenic tumor [18]. Calycosin can cause stress-induced phosphoprotein 1 (StIP1) expression increase. StIP1 as a crucial chaperone has the possibility to substantial accommodation of cytogenesis in tumor, stress reaction, and cell proliferation and differentiation [19]. The expression of StIP1 is obviously downregulated in paclitaxel-resisted ovarian cancer cell strain OC3/Tax300 [20]. Calycosin can also increase two antioxidant proteins which have functions in antitumor. Peroxiredoxin, belonging to antioxidant protein superfamily involved in cell proliferation and differentiation, enhances activity of NK cell and protects free radical sensitive protein and DNA normal replication. The downregulation expression of Pdx1 was closely related to the balance between oncogene and antioncogene destroyed and the invasiveness of giant cell tumor of bone (GCTB) [21]. Biliverdin reductase B was increased by calycosin and produced bilirubin which has protection function for organism. We also found two proteins which are crucial for cancer cell growth expression inhibited by calycosin. Thioredoxin peroxidase B (Prx II) is a novel inhibitor of apoptosis of cancer cells [22]. They efficiently reduced the intracellular level of H_2_O_2_ produced stimulated by various cell surface ligands. The peroxiredoxins family was reported to be closely related to various causes of liver fibrosis. They were found to be upregulated in liver fibrosis caused by chemical induction. Prx II has shown a significant upregulation at the middle-stage fibrosis and played an important role in hepatocarcinogenesis [23]. Research showed that the hepatocellular carcinoma cells proliferation and clone formation decreased and cell apoptosis was enhanced obviously when Prx II expression was inhibited [24]. Prx II also has shown a downregulation in the hepatocellular carcinoma cells after being treated with calycosin, which might be related to the antitumor mechanism of calycosin. We found that calycosin can inhibit thioredoxin peroxidase B protein expression. High-mobility group box 1 protein (HMGB1), a chromatin-associated nuclear protein and extracellular-damage-associated molecular pattern molecule (DAMP), is an evolutionarily ancient and critical regulator of cell death and survival. Over-expression of HMGB1 is associated with each of the hallmarks of cancer including unlimited replicative potential, angiogenesis, evasion of apoptosis, self-sufficiency in growth signals, and insensitivity to inhibitors of growth. In our study, calycosin downregulated HMGB1, which may be related to the antitumor mechanism of calycosin on hepatocellular carcinoma cells.

In conclusion, calycosin can inhibit cell growth of BEL-7402 by blocking cell-cycle and regulating some tumor genes translation. The cell-cycle-related genes which we found only in RNA array chip but not in the 2D gel maybe because the expression quantity is limited. The molecular mechanism was related to downregulated expression of TFDP-1, SKP2, and CDKN2D and upregulated expression of CDC2, CDK7, and CCNB1. And then we discovered certain proteins related to antitumor including transgelin 2, PNPO, StIP1, PDX 1, biliverdin reductase B, HMGB1, and Prx II which were changed after calycosin treatment. The functions of differentially expressed proteins may have a possibility to be involved in antitumor mechanism of calycosin.

## Figures and Tables

**Table 1:** The effect of calycosin on proliferation of BEL-7402 cells ($\overset{-}{x}\pm s$).

Calycosin concentration (mmol/L)	OD value	Inhibition rate %
Control (0.5% DMSO)	0.421 ± 0.045	0.5
0.004	0.410 ± 0.036	3.1
0.111	0.306 ± 0.013**	27.7
0.352	0.156 ± 0.042**	63.1
1.113	0.084 ± 0.021**	80.1

Data expressed as mean $\bar{x}\pm s$, **P < 0.01 compared with control.

**Table 2:** The effect of calycosin on cell cycle of BEL-7402 cells ($\bar{x}\pm s$).

Group	G_1_ Stage (%)	S Stage (%)	G_2_/M Stage (%)
Control (0.5% DMSO)	63.31 ± 0.99	28.84 ± 0.82	7.86 ± 0.67
Calycosin	74.27 ± 2.31**	12.94 ± 4.53**	12.79 ± 2.28**

Data expressed as mean $\bar{x}\pm s$, **means *P* < 0.01 compared with control.

**Table 3:** Isoelectric point and molecular weight of the differentially expressed proteins of human hepatoma carcinoma cells after treatment by calycosin.

Spot	PI	Mr (kD)	Expression
2	5.44	21795	↓
8	9.74	19617	↓
9	5.80	22708	↑
10	6.77	28975	↑
21	8.44	24400	↑
22	7.42	20913	↑
23	8.27	22096	↑
26	6.41	18964	—
31	6.61	29698	↑
32	6.62	29969	↑
37	7.81	68037	↑
42	5.42	19641	↑
52	6.77	28975	↑
58	6.67	28786	↑
3	8.15	65999	↑
34	5.94	22332	↑
61	7.13	22105	↑
33	6.62	29969	—

PI represents isoelectric point; Mr represents molecular weight; “↑” represents the upregulated protein; “↓” represents the downregulated protein; “—” represents the disappeared protein.

**Table 4:** Differentially expressed proteins of human hepatoma carcinoma cells after treatment by calycosin.

Spot	Protein name	NCB Inr ID no.	Sequence coverage (%)	Score	Protein expression
2	Thioredoxin peroxidase B	gi∣9955007	69	187	↓
3	Keratin 1	gi∣119395750	50	159	↑
8	High-mobility group box 1	gi∣119628863	48	80	↓
21	Transgelin 2	gi∣4507357	88	483	↑
22	Neuropolypeptide h3	gi∣913159	74	102	↑
23	Peroxiredoxin 1	gi∣4505591	79	220	↑
31	LIM and SH3 protein 1	gi∣5453710	69	98	↑
32	Pyridoxine 5′-phosphate oxidase	gi∣8922498	41	78	↑
34	Abhydrolase domain containing 14B	gi∣14249382	58	233	↑
37	Stress-induced phosphoprotein 1 (Hsp70/Hsp90-organizing protein) isoform 1	gi∣114638255	67	180	↑
42	Nonmetastatic cells 1, protein (NM23A) expressed in isoform a	gi∣38045913	63	77	↑
52	Endoplasmic reticulum protein 29 isoform 1 precursor	gi∣5803013	61	129	↑
58	Phosphoglycerate mutase 1 (brain)	gi∣119570326	61	135	↑
61	Biliverdin reductase B	gi∣4502419	70	219	↑

“↑” represents the upregulated protein; “↓” represents the downregulated protein.
